# Otology

**Published:** 1894-03

**Authors:** W. H. Harsant


### OTOLOGY.

In the year 1887 I reported in this *Journal*[1] a case of septic thrombosis and phlebitis of lateral sinus following middle-ear disease, which went on to a fatal termination, as all cases of the kind did at that period. But in the preceding year Mr. Victor Horsley had made the valuable suggestion[2] that ligature of the internal jugular vein might arrest the flow of septic material into the blood; and in April, 1889, Mr. Arbuthnot Lane[3] reported a case of purulent thrombosis of lateral sinus in a boy aged 10, in whom he ligatured the internal jugular vein, and the boy made a rapid recovery.

In 1890 Mr. Ballance[4] read a paper before the Medical Society of London, wherein he recorded four successful cases of this kind, accompanied by some very valuable rules and suggestions as to the management of this hitherto very fatal class of cases. Since that time, many other surgeons—especially Macewen and Barker—have recorded cases and formulated rules, so that at the present time our knowledge of the symptoms and our method of dealing with septic thrombosis of the lateral sinus are as perfect as in any other surgical malady that may be named. The literature of the subject has been somewhat extensive during the past year, but I have selected the three following cases as among the most instructive.

Dr. John L. Adams, of New York, describes a case[5] of thrombosis of the lateral sinus where Ballance's operation was performed by Dr. Abbé. The patient, a girl aged 20, had suffered from a purulent discharge from the right ear two years previously, followed by continuous slight discharge until four weeks before the present illness, when it entirely ceased. Seventeen days before admission she began to have headaches, and ten days ago the right ear and right side of the head began to be painful. Repeated attacks of shivering and vomiting followed, and the head-pains became very severe. On examination, a perforation was found in the right drum-membrane. There was marked mastoid tenderness, with some induration, and a small amount of pus was found in the external auditory canal. Pain was experienced at back of neck upon moving the head, but there was no œdema of neck or in region of jugular vein. The temperature was 101.2° and the pulse 76. The mastoid antrum was first opened and a free communication established with the drum cavity. The symptoms became more severe next day, and the temperature reached 103.5°. Convergent squint and paresis of sixth nerve were noticed. The rigors were intensified, and the patient became drowsy and stupid.

The diagnosis of cerebral abscess was then made, and a

[1] Vol. v., p. 37. [2] *Tr. Clin. Soc.*, vol. xix., p. 290. [3] *Ibid.*, vol. xxii., p. 260.

[4] *Lancet*, 1890, vol. i., p. 1057. [5] *Arch. Otol.*, vol. xxii., no. 2.

trephine-hole over the temporo-sphenoidal lobe was established, through which needles were thrust into the brain substance without finding pus. The lateral sinus was then opened by another trephine-hole, and the sinus was found slightly pulsating, thickened, and inflamed. The internal jugular vein was ligated in the neck, after which the foul septic thrombus was washed out of the lateral sinus. The patient did not recover, and an autopsy revealed circumscribed suppurating meningitis resulting from middle-ear disease. The operation would probably have been successful had it been performed earlier.

Messrs. Bernard Scott and Arbuthnot Lane report a case[1] of subdural abscess in posterior fossa, with septic thrombosis of lateral sinus, treated successfully by a similar operation. The patient had suffered from otitis media for four years. At intervals there were attacks of pain in the ear and in the mastoid process and its vicinity, together with headache on the same side of the head. The attacks of pain increased in severity until May 29th, when the patient became suddenly worse and had a rigor. On the 31st there was another rigor, the temperature rising to 104.5°. Mr. Scott diagnosed septic thrombosis and called in Mr. Lane, who operated on June 2nd. Some small cancellated spaces filled with pus were found in the mastoid process, and the antrum was enlarged and filled with foul-smelling pus. On freely exposing the dura mater of the posterior fossa, a large subdural abscess was opened. This abscess extended over the lateral sinus for about an inch and a-half; the wall of the sinus was, like that of the adjacent dura mater, much inflamed. Bone was freely removed with the gouge, until the sinus was fully exposed for more than two inches. The internal jugular vein was then tied in the neck, and the wound closed accurately with a continuous suture. The inflamed wall of the lateral sinus was cut away with scissors up to the torcular margin, when a large conical clot about an inch and three-quarters in length was pulled out of the sinus and internal jugular vein, into which it extended. Bleeding was stopped by plugs of iodoform gauze, and the contents of the middle ear were removed by a curette. Recovery was uninterrupted.

Dr. Urban Pritchard and Mr. G. L. Cheatle also report a successful case of this operation.[2] The patient was a girl, aged 13, who had suffered from purulent discharge from the ears during infancy and early childhood, but had been free from any ear-trouble for several years. Eight days before being seen, earache and headache on the left side came on without obvious cause, accompanied with pain in the left side of the neck on movement. These pains gradually increased in severity, became constant and agonising, and prevented sleep. Vomiting was an early and distressing symptom. Rigors developed—sometimes two or three a day,—and twitchings of the right leg were said to

[1] *Lancet*, 1893, vol. i., p. 138. [2] *Ibid.*, p. 471.

have been noticed during sleep. The patient was restless, but answered questions quite reasonably and connectedly. There was no redness, swelling, or œdema of or about the ear, but percussion two inches and a-half behind the pinna gave rise to pain. The upper part of the neck was somewhat swollen, but fluctuation could not be detected. The temperature was 102°, pulse 116, respirations 44.

Mr. Cheatle decided to operate upon the following lines: (1) To chisel into the mastoid antrum; (2) if necessary, to open up the mastoid cells; (3) if nothing to account for the symptoms could then be found, to chisel backwards and open up the lateral sinus. On the first introduction of the chisel into the antrum, pus bubbled out with the blood. The opening thus made was enlarged and a few streaks of pus were evacuated. The bone-opening was next enlarged upwards and backwards, and an exploratory needle passed into the brain substance without result. The lateral sinus was then exposed and found to be blocked with a fœtid clot; so the internal jugular vein was divided between two ligatures, and the fœtid clot scraped out from the lateral sinus which was syringed with perchloride of mercury solution.

Three days afterwards, in consequence of a continued elevation of temperature, the lateral sinus was still further scraped out and its outer wall completely removed; the upper part of the jugular vein was likewise dissected out, after which the patient made an excellent recovery.

W. H. Harsant.

